# Geroprotective and senoremediative strategies to reduce the comorbidity, infection rates, severity, and lethality in gerophilic and gerolavic infections

**DOI:** 10.18632/aging.102988

**Published:** 2020-03-31

**Authors:** Alex Zhavoronkov

**Affiliations:** 1 Insilico Medicine, Hong Kong Science and Technology Park (HKSTP), Tai Po, Hong Kong; 2 The Biogerontology Research Foundation, London, UK

**Keywords:** COVID-19, SARS-CoV-2, coronavirus, sirolimus, rapalog

## Abstract

The recently identified SARS-CoV-2 betacoronavirus responsible for the COVID-19 pandemic has uncovered the age-associated vulnerability in the burden of disease and put aging research in the spotlight. The limited data available indicates that COVID-19 should be referred to as a gerolavic (from Greek, géros “old man” and epilavís, “harmful”) infection because the infection rates, severity, and lethality are substantially higher in the population aged 60 and older. This is primarily due to comorbidity but may be partially due to immunosenescence, decreased immune function in the elderly, and general loss of function, fitness, and increased frailty associated with aging. Immunosenescence is a major factor affecting vaccination response, as well as the severity and lethality of infectious diseases. While vaccination reduces infection rates, and therapeutic interventions reduce the severity and lethality of infections, these interventions have limitations. Previous studies showed that postulated geroprotectors, such as sirolimus (rapamycin) and its close derivative rapalog everolimus (RAD001), decreased infection rates in a small sample of elderly patients. This article presents a review of the limited literature available on geroprotective and senoremediative interventions that may be investigated to decrease the disease burden of gerolavic infections. This article also highlights a need for rigorous clinical validation of deep aging clocks as surrogate markers of biological age. These could be used to assess the need for, and efficacy of, geroprotective and senoremediative interventions and provide better protection for elderly populations from gerolavic infections. This article does not represent medical advice and the medications described are not yet licensed or recommended as immune system boosters, as they have not undergone clinical evaluation for this purpose.

## INTRODUCTION

Aging is a complex, multifactorial process [1] that leads to loss of function and is the primary risk factor for major human pathologies including cancer, diabetes, cardiovascular disorders, and neurodegenerative diseases [1, 2]. Although there is still much debate in the scientific community, proposals have been made to classify aging as a disease in order to develop therapeutic strategies to prevent or delay the onset of age-related illnessess [3–5]. Increasing frailty with age leads to an increased risk of many diseases. These diseases are commonly referred to as age-related [6]. Many pathogens are more infectious and prevalent in the elderly, [7–10] and may be referred to as gerophilic (from Greek, *géros* “old man” and *philia*, “love”). Some infections, including COVID-19, are not exclusively gerophilic, as younger people may also become infected. However, these individuals have mild symptoms or remain asymptomatic, while the elderly experience substantially more severe symptoms and lethality. The term gerolavic (from Greek, *géros* “old man”, and *epilavís*, “harmful”) may more appropriately describe infections that cause the most harm in the elderly.

### COVID-19 is a gerophilic and gerolavic infection

**Figure 1:**
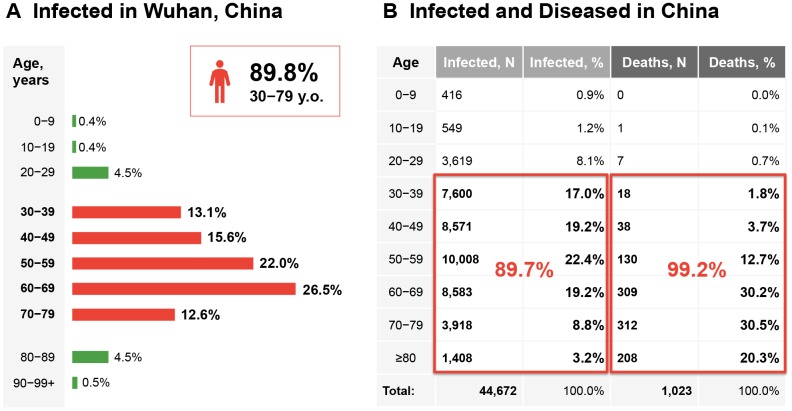
**COVID-19 as a gerophilic and gerolavic infection.** (**A**) Distribution of patients diagnosed in the city of Wuhan only through February 11, 2020. (**B**) Age distribution of the infected and diseased patients in Mainland China through February 11, 2020. The figures are adopted and generated from [12] (http://weekly.chinacdc.cn/en/article/id/e53946e2-c6c4-41e9-9a9b-fea8db1a8f51).

Statistics from the COVID-19 pandemic indicate that COVID-19 is a gerolavic infection, one that disproportionately affects the elderly (Figure 1). The majority of the infected population are 50 and older, while the majority of the deceased are 60 and older [11]. At the same time, fewer than 10% of the infectedwere 30 or younger, while mortality rates are 2.8% in males versus 1.7% in females [12]. However, these values for the young are skewed upwards because of higher mortality among the elderly. According to Worldometers [13], an online resource aggregating data on COVID-19, of the 139,580 people infected worldwide as of March 13, 2020, 70,733 patients had recovered and 5,120 had died. Based on these data, the mortality rates (number of deaths/number of cases), or the probability of dying if infected by the virus, were determined to be 3.6% for individuals aged 60-69, 8% for individuals aged 70-79, and 14.8% for patients aged 80 years or older. An open coronavirus analysis project by the Nobel Laureate Michael Levitt [14] and a recent study by Mizumoto et al. [15] provide further insight into the mortality rates of COVID-19, specifically using data from the Diamond Princess Cruise, where all passengers were exposed to SARS-CoV-2 for an extended period. Of the approximately1,690 passengers over 65 years of age, 7 passengers died, suggesting a death rate of 0.41%. This death rate is approximately 4.3 times higher than that of influenza. As more countries start reporting statistics, these death rates are likely to be adjusted. These statistics indicate that the infectivity of SARS-CoV-2, and the severity and lethality of COVID-19, are age-related.

### The challenges of assessing the burden of gerolavic epidemics

The online resources Our World in Data [16] and The Lancet’s Global Burden of Disease [17] provide deep visual insight into the global burden of disease by cause and demographics. In 2017, there were 56 million deaths globally; over two-thirds of these (76%) were in people over 50 years of age. According to the online period life tables put out by the US Social Security Administration [18], the annual chance of death in 2015 (the probability of dying within one year) for a person over 80 was 5.2%, increasing to 14.8% by the age 89.

According to estimates by the US Centers for Disease Control [19], approximately 5,945,690 individuals older than 65 had symptomatic influenza during the 2017-2018 season, resulting in 3,329,586 medical visits, 540,517 hospitalizations, and 50,903 deaths. Hence, the death rate for those hospitalized with influenza was 9.4% for patients over 65.

However, many of the COVID-19 patients over 65 years have one or more comorbities [20], and it is often difficult to attribute the cause of death exclusively to the gerolavic coronavirus. Currently, there are no accurate statistics linking smoking status, lifestyle, and behavior to the severity and lethality of COVID-19. Despite this, it is possible that these factors, as well as frailty and comorbidities, play a substantial role. Gerolavic diseases such as COVID-19 may not significantly increase the yearly death rates for each individual age group; however, these diseases substantially accelerate death from multiple conditions, and compress the process to less than two weeks. Comorbidity is also the likely cause of the substantial differences in death rates among different countries due to differences in patient demographics, levels of preparedness, when the epidemic began locally, and reporting [13]. Only the data available from China, where the epidemic has subsided, and the Diamond Princess cruise ship were used for this study.

### Aging and immunosenescence

One of the possible causes of the age-associated increases in COVID-19 infection rate, severity, and lethality is immunosenescence. Immunosenescence is a well-known age-related process contributing to the global burden of disease [21]. It is among the major factors underlying the difference between younger and older populations in the response rate to vaccinations and the virulence of infectious diseases [22–24]. Among the factors contributing to immunosenescence is the chronic involution of the thymus gland with increased age. Indeed, the infection rates of COVID-19, separated by age, are correlated with involution of the thymus [12]. The thymus gland is most active early in life, reaching maximum size within the first year. Its activity then declines with age until an individual reaches 40 to 50, after which there are negligible traces of the thymus remaining, replaced by fibrotic tissue [25]. As a result of thymic involution, the number of naïve T cells exiting the thymus decreases significantly, with substantial declines in older age [26].

**Figure 2:**
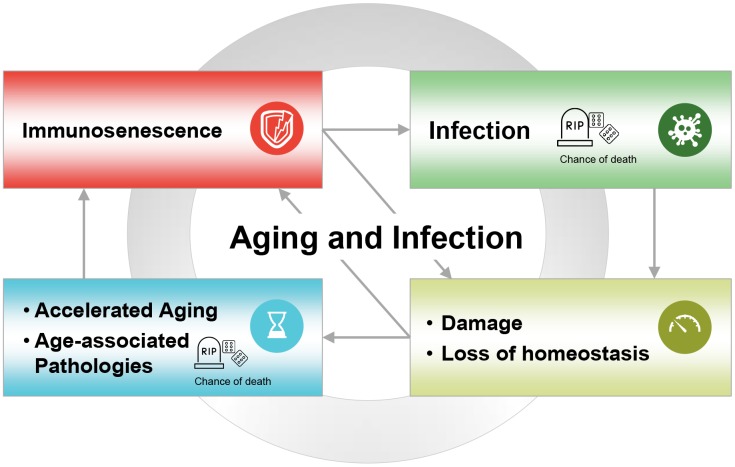
**The vicious circle of aging and infection, where age-associated immunosenescence leads to reduced ability to resist infection; infection leads to increased damage, loss of homeostasis, and accelerated aging; which in turn leads to age-related diseases, further accelerating immunosenescence.** Infections and other age-related diseases are among the main causes of death in the developed world and developing countries.

Besides thymic involution, there are many other factors driving immunosenescence and the increase in multimorbidity that occurs during aging [22, 27, 28]. Figure 2 illustrates the hypothesised reciprocal relationship between immunosenescence and infectious disease acquisition. In this model, age-associated immunosenescence leads to a reduced ability to resist infection, while infection produces biological damage and loss of homeostasis. This ultimately contributes to accelerated aging and the development of age-related diseases, and further accelerates immunosenescence. In support of this model, infections and other age-related diseases are among the main causes of death in the developed world and in developing countries.

### Addressing a gerolavic virus: classical versus geroprotective and senoremediative strategies

Due to the gerolavic nature of COVID-19, the classical preventative measures and treatment strategies used for targeting infectious diseases may not be as effective, and there is a need for alternative geroprotective and senoremediative strategies. There are multiple clinical trials in progress using established medical interventions to treat COVID-19, with the number of studies rapidly increasing [23]. For a list of promising SARS-CoV-2/COVID-19 targets and treatment approaches, please refer to the Global Health Drug Discovery Institute’s portal dedicated to COVID-19 [29].

Here we compare the expected benefit of treatments for elderly populations (60 years and older) that are currently in development, including standard preventative strategies such as vaccines and antivirals targeting SARS-CoV-2, and the potential added benefit of speculative geroprotective strategies such as rapalogs, NAD+ boosters, senolytics, and stem cell treatment. These additional measures may be used in isolation or as adjuvant therapies to reduce infection risk, symptom severity, or improve vaccine efficacy.

### Vaccines

Vaccine development is one of the most successful approaches for combating viral diseases globally, and is often regarded as one of the greatest advances in biomedical science and integrated healthcare. Currently, there are around 60 active clinical trials related to a SARS-CoV-2 vaccine, most of them taking place in China [30]. Broadly speaking, the success of a vaccine partly depends on the similarity of the vaccine strain with the viral pathogenic strain in question. In addition, an individual’s immune response must be sufficiently strong to mount a reaction to the vaccine that can later confer protection against the pathogen, should exposure occur. Our current strategy for targeting annual influenza viral outbreaks focuses on effective vaccination based on predictions of strain variants. People >60 years of age with chronic medical conditions, such as type 2 diabetes or cardiovascular disease, direct immunosuppression from HIV, post-transplant or biologic treatment, pregnant individuals, or those with BMI>40, are believed to be at higher risk for influenza infection due to a weakened immune response [31]. Similarly, vaccines do not provide complete protection in older populations due to age-related declines in immune function and accumulation of multi-morbidities. Outbreaks can occur in elderly nursing homes even when vaccination rates reach 80-98% uptake [32]. Thus, even when a successful vaccine for SARS-CoV-2 becomes available, a geroprotective agent might be used in combination with the vaccine to boost the immune response. Currently in most countries, the influenza vaccine formulation is determined 6-9 months before the expected outbreak season and the strains are based on the precedent season’s viruses. As a result, vaccine efficacy is expected to differ from season to season. Thus, an ongoing additive geroprotective therapy is of high importance [33, 34] and is applicable beyond the current pandemic. While vaccines may be the best preventative strategy for reducing the infection rates, severity, and lethality of COVID-19, the rates to vaccines in the elderly will likely be lower [35] and vaccine potentiation strategies [36] may be explored and evaluated in clinical trials.

### Chemoprophylactic and therapeutic therapies

While chemoprophylaxis is not routinely indicated and is not considered a replacement for vaccination, using influenza as an example, prophylactic treatment prior to symptom onset in high-risk groups or after close contact exposure to the virus is an alternative preventative strategy against viral disease [31]. For influenza, the neuraminidase inhibitors oseltamivir and zanamivir are occasionally given prophylactically to high-risk individuals in long-term care facilities during outbreaks [37]. Nevertheless, there is currently no definitive benefit proven for antiviral treatment outside of these specific circumstances, as it comes at a cost and may be associated with side effects; for example, zanamivir can induce bronchospasms in patients with chronic respiratory disease and asthma. Pharmacotherapy for individuals with infection remains the cornerstone of clinical practice. The success of antiviral treatment is condition-specific, ranging from new, direct-acting antiviral drugs that offer a potential cure for hepatitis C [38]; to the highly active antiretroviral drugs that enable HIV positive individuals the prospect of a healthy life expectancy while on treatment; to antiviral drugs for herpes simplex types 1 and 2 that lead to symptom alleviation but do not eradicate the latent infection; to antivirals for seasonal influenza that are believed to reduce symptom duration, and reduce complications and transmission risk.

Other anti-influenza medications licensed for treatment, aside from oseltamivir and zanamivir, consist of an intravenous neuraminidase inhibitor, peravamir, and a novel oral inhibitor of cap-dependent endonuclease, baloxavir. Neuraminidase inhibitors are effective against both influenza A and B, while an additional class of antivirals that are no longer recommended for treatment of influenza due to reduced efficacy, neurological side effects, and widespread resistance, adamantanes (M2 inhibitors, amantadine and rimantadine), are only active against influenza A [31]. Although many patients with influenza exhibit minimal clinical improvement upon treatment with these medications, they are currently recommended for treatment of all hospitalised patients, even prior to laboratory confirmation of influenza infection. Evidence shows that the greatest benefit is seen when these drugs are administered 24-30 hours prior to symptom onset, in which case they reduce symptom duration by 0.5-3 days and reduce transmission risk [39–42].

### Symptomatic treatments

According to the recent COVID-19 treatment guidelines in China [43], symptomatic treatment for COVID-19 patients is recommended for mild cases and consists of rest, isolation, adequate hydration, analgesia, and antipyretic medication. Moderate and severe cases (mostly hospitalized) require additional measures, such as careful fluid balance, intravenous antibiotics for superinfections, oxygen supplementation, non-invasive ventilation with or without positive pulmonary pressure, and in some cases intubation and mechanical ventilation. Although the projected global infection rates are variable, we share a common concern that outside of China there may be an insufficient number of beds for hospitalization and ventilation units if the disease spread does not slow down.

### COVID-19 rehabilitation

Even asymptomatic COVID-19 infections can induce lung fibrosis, which may lead to reduced function of the respiratory system. Further, severe cases are often complicated by bacterial infections and pneumonia, leading to fibrosis. Therefore, COVID-19 rehabilitation may include antifibrotic compounds, anti-COPD, and regenerative medicine therapies.

### Geroprotective and senoremediative strategies for COVID-19 gerolavic infection

There are multiple interventions proposed in the academic literature to remedy age-associated increases in infection rates, severity, and lethality for a variety of infections. For example, regular increased physical activity has been proposed to reduce immunosenescence [44]. Fahy et al. [45] and Horvath [46] have suggested that a combination of the potentially geroprotective compound metformin, recombinant human Growth Hormone (rhGH), and dehydroepiandrosterone (DHEA) may reverse biological age, as measured using the methylation aging clock, and immunosenescence [45]. Geroprotectors were previously proposed to enhance human radioresistance in extreme conditions [47]. While there is no clinical evidence yet suggesting age reversal or improved immune function in the elderly, efforts are being made to identify new geroprotectors using human data and artificial intelligence [48–50]. Further, the use of natural compounds that mimic the effects of known geroprotectors is generally recognized as safe [51]. However, attempts have been made to develop criteria for the evaluation of geroprotectors for clinical validation.

**Figure 3:**
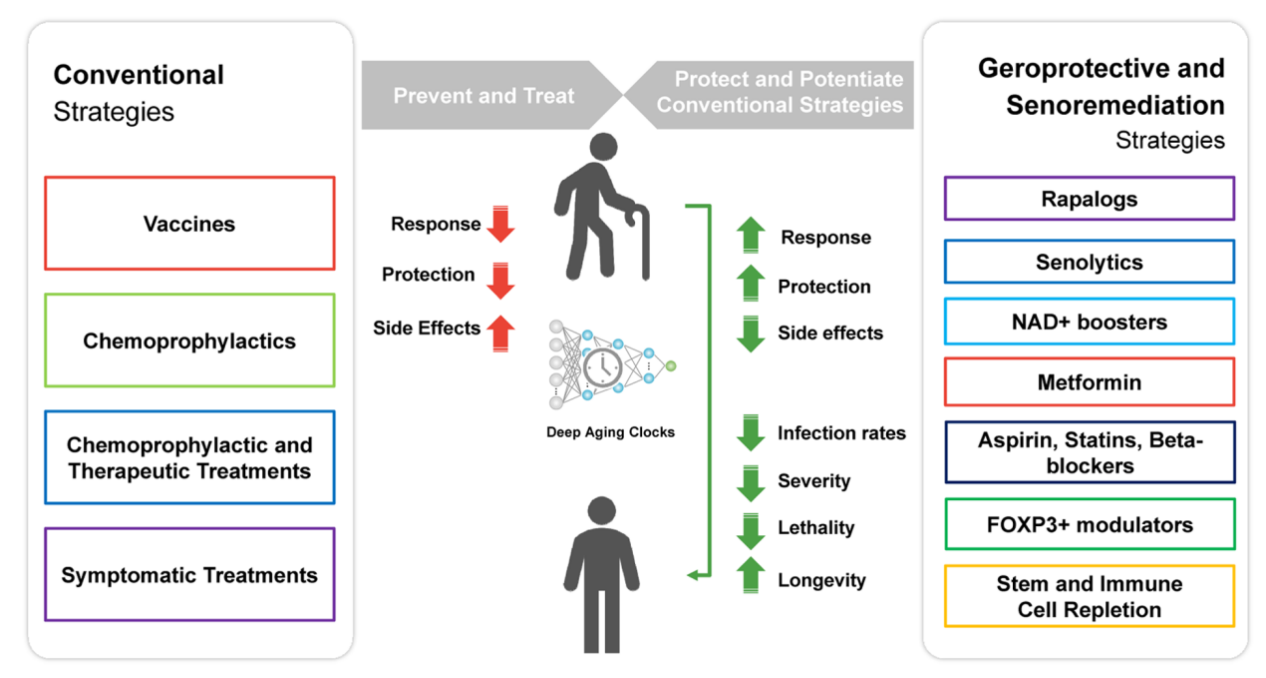
**Geroprotective and senoremediative strategies, such as a course of low-dose rapamycin, may potentiate the response to conventional prevention and treatment strategies, prevent infection, reduce disease severity and lethality, and may also increase longevity.**

There are multiple strategies proposed to restore immune function in the elderly [52], and multiple databases of geroprotectors exist [53, 54]. However, to date the only known geroprotectors backed by promising clinical evidence of improved immune response to viral infection in the elderly, although still limited by a lack of large clinical trials, are sirolimus (rapamycin) and everolimus. These may be used as single agents in combination with other treatments (Figure 3).

### Rapamycin and rapalogs

Sirolimus (rapamycin) is a well-known geroprotector, known to effectively increase lifespan and slow aging in many species, including yeast [55, 56], *Drosophila* [57, 58], *C. elegans* [59], and mice [60–64]. It also delays age-related diseases in humans [65–68], and Blagosklonny proposed rapamycin for the prevention of multiple age-related diseases in humans [69–72].

Sirolimus and rapalogs are commonly used as immunosuppressants. Rapalogs, the derivatives and mimetics of rapamycin, target critical factors in the rapamycin (TOR) pathway. Everolimus (RAD001), another close structural derivative of sirolimus developed by Novartis, acts as an immunosuppressant; but like sirolimus, it has many other properties beyond immunosuppression [73]. Paradoxically, these compounds also exert immunostimulatory effects, such as boosting T cell responses in reaction to pathogen infection and vaccination [74]. Nevertheless, this would not be the first case of a physiological paradox in clinical medicine. The administration of beta-blockers to heart failure patients at first seemed contradictory, as these compounds slow down an already failing heart, but proved to provide the most benefit for the treatment of heart failure patients. Likewise, hormonal treatment of hormone-dependent cancers, such as testosterone-dependent prostate cancer, seems incongruous. However, administration of a synthetic version of gonadotropin-releasing hormone (GnRH) in a different dosing regime from the cyclical secretion that occurs physiologically, which normally indirectly increases testosterone levels, actually reduces hormone levels. Therefore, it might be possible that a drug that is known to be an immunosuppressant might in a different dosing regimen prove to be an immunostimulant. However, extremely cautious clinical validation is required as this treatment might carry significant risks; indeed, there is some indication that morbidity from coronavirus infections occurs from secondary overactive immune responses [75, 76]. In addition to rapamycin, other agents that inhibit mTOR, such as Torin1, Torin2, AZD8055, PP242, KU-006379 and GSK1059615, may act similarly to rapamycin in low-doses and may have a geroprotective effect [77–79]. Substantial pre-clinical validation would be required to apply these compounds to specific age-associated diseases and to explore clinical applications of these compounds in human clinical trials.

Multiple clinical observations suggested that patients with cytomegalovirus (CMV) disease who were treated with rapamycin demonstrated better outcomes and were better able to control CMV viremia than patients treated with standard calcineurin inhibitor-based immunosuppression following transplantation [74, 80]. In 2009, two seminal studies of sirolimus demonstrated the immunostimulatory effects of rapamycin on the CD8+ memory T cell response following pathogen infection [74, 80]. Later studies also showed that monkeys treated with sirolimus exhibited increased recall responses and enhanced differentiation of memory T cells following vaccination with Modified Vaccinia Ankara [81].

Additional clinical studies by Mannick et al. [82, 83] demonstrated the immunostimulatory role of rapalogs in the elderly using the Novartis rapalog everolimus (RAD001), a close structural analog of sirolimus (rapamycin). Administration of everolimus ameliorated immunosenescence in healthy elderly volunteers and enhanced the response to the influenza vaccine by around 20% at doses that were well tolerated [82]. Further studies demonstrated enhanced immune function and reduced infection in elderly patients receiving tolerable doses of everolimus. Mannick et al. also conducted a phase 2a randomized, placebo-controlled clinical trial which demonstrated that a low-dose combination of dactolisib (BEZ235) and everolimus in an elderly population was safe and associated with a significant (P=0.001) decrease in the rate of reported infections [83].

Mannick and colleagues further conducted a phase 2a randomized, placebo-controlled clinical trial that demonstrated that a low-dose combination of dactolisib (BEZ235), a PI3K inhibitor [84] and catalytic mTOR inhibitor, and everolimus in an elderly population was safe and associated with a significant (P=0.001) decrease in the rate of reported infections [83]. A follow-up trial of dactolisib alone (BEZ235 rebranded as RTB101) for prevention of respiratory tract infections in the elderly did not meet the primary endpoint and further trials were withdrawn [85]. In prior studies, everolimus (RAD001) was used as a standalone agent or in combination with dactolisib, which may explain the phase 3 failure of BEZ235/RTB101. There are over 95 phase 3 and phase 4 studies for these agents [86], and they are generally well tolerated even in high doses. Even though it may not be commercially viable due to the patent expirations, clinical trials should be conducted to evaluate the effectiveness of these agents for protection against SARS-CoV-2 (COVID-19) and other gerophilic and gerolavic infections.

### Metformin

Metformin is a drug approved to treat type 2 diabetes but appears to target a number of aging-related mechanisms, including decreasing IGF-1 levels, inhibiting mTOR, and inhibiting mitochondrial complex 1. Metformin is currently in the first large-scale human clinical trial of aging, the Targeting Aging with Metformin (TAME) study, which is investigating its effect on time to a new occurrence of a composite outcome that includes cardiovascular events, cancer, dementia, and mortality [87]. Metformin would likely still be contraindicated in elderly patients with advanced chronic kidney disease and eGFR<15. A reduced dose would potentially be required for eGFR<30 due to a risk of lactic acidosis. The effects on gerophilic and gerolavic infections should be carefully examined in the context of the TAME study, and other clinical trials involving metformin.

### NAD boosters

Nicotine adenine dinucleotide (NAD) is a cofactor of multiple fundamental enzymes. It is involved in metabolic regulation through the Krebs (citric acid) cycle, oxidative phosphorylation, and cellular signaling, as well as cellular senescence and DNA repair through the poly-ADP-ribose polymerases (PARPs), sirtuins, and CD38. NAD levels decrease with aging, and benefits of NAD supplementation have been reported in multiple animal studies. Although no proof of a similar effect in humans has been shown, several clinical trials are in progress [88–91]. Supplementation with nicotinamide riboside (NR) in one human study produced an improvement in exercise capacity in a population with a mean age of 71 [92]. This compound was also shown to reduce blood pressure in hypertensive patients [93].

Nicotinic acid is another NAD precursor that is converted in the body to NAD by the enzymes NAPRT, NMNAT, and NADS. Large-scale trials of nicotinic acid for cardiovascular disease [94, 95] showed some efficacy, but produced adverse side effects, such as headache, skin flushing, and dizziness [96].

NAD acts at a cellular level and it is still unclear whether oral or intravenous supplementation with NAD donors, such as NR and nicotinic acid, will increase NAD levels and exert a clinical benefit in humans. However, COVID-19 patients may benefit tremendously from these compounds, as SARS-CoV-2-infected patients have increased levels of CD38+, and NAD has been shown to enhance DNA repair via PARP pathways [97].

Caution should be exercised when conducting any clinical trials for NAD boosters against gerophilic and gerolavic infections, as the underlying biology of NAS metabolism and viral infections is still poorly understood. Recent studies in humans demonstrate that NR supplementation reduces the levels of circulating inflammatory cytokines [98], while Nicotinamide Mononucleotide (NMN) may reduce the expression of these cytokines [99]. Other studies implicate NAD in increased cytokine production [100] and the NAD+-consuming enzyme CD38 in increased inflammation [101]. Additional immunological studies of NAD boosters must be performed before clinical trials may be conducted. However, considering the large consumer base of NR and NMN supplements, it may be possible to conduct metastudies on influenza and SARS-CoV-2 infectivity, severity, and lethality.

### Senolytics

Senolytics are drugs that are postulated to selectively destroy senescent cells, which accumulate with aging and exhibit senescence-associated secretory phenotype (SASP), through senolysis, apoptosis, immunosurveillance, or other mechanisms of action [102]. SASP is now hypothesised to lead to NAD depletion and thus initiate or perpetuate an increase in sterile chronic inflammation. Many drug classes, ranging from fibrates to cardiac glycosides, have been reported to have senolytic properties in animal models [103]. However, recent promising human data have been reported with the tyrosine kinase inhibitor dasatinib in combination with the plant flavonol quercetin in a trial by the Mayo Clinic [104]; flavonoid polyphenols have also proven beneficial. In addition, pre-clinical and clinical data suggest that flavonoids may be used for prophylaxis in upper respiratory tract infections [105].

Although senolytic drugs would have a scientifically plausible role in biological age reversal and thus reduction of mortality from gerolavic viruses like SARS-CoV-2, it has not been shown that these classes of drugs would protect against infection or could be used as adjuncts to vaccination. In addition, there remains the risk that senolytics would not be sufficiently specific to discriminate between deleterious senescent cells and quiescent (dormant) cells, which might still differentiate into the mature cell types of a given tissue, and could thus deplete beneficial protective stem cell reserves.

### Intermittent caloric restriction

It has been shown in multiple studies that calorie restriction leads to increased lifespan and improved cardiometabolic markers, even when initiated in middle age [106]. Caloric restriction should be considered as a preventive measure on a long-term basis and is indicated for younger individuals. Some elderly patients already have frailty syndromes and evident sarcopenia/ osteopenia, which limits the suitability of intermittent caloric restriction. Nevertheless, the benefits of time-restricted feeding and intermittent fasting go beyond simple caloric restriction due to the production of ketones. Ketones are active signaling molecules that play a major role in the PPAR, sirtuin, NAD and CD38 pathways, encourage autophagy (the removal of damaged cellular materials), modulate the immune response, and have been explored in clinical trials as an adjuvant therapy for cancer treatments [107]. Within 8-12 hours of food restriction, ketones are believed to rise to 0.2 to 0.5mM and continue to increase within the first 48 hours to 1 to 2mM [108]. Under fasting conditions the major body ketone in the plasma, beta-hydroxybutyrate (BHB), increases. BHB is believed to confer the major metabolic benefit of fasting and is in development as an independent therapeutic supplement.

### T cell activation

An age-related decrease of thymic function consequently reduces the levels of specific T cell subsets [109]. FOXP3+ regulatory T (Treg) cells are critical in homeostasis of the immune system and are believed to start declining in numbers at around 50-60 years of age; this remains one of the fundamental drivers of immunosenescence. There are two known origins for Treg cells: thymus-derived Treg cells and peripherally-derived Treg (pTreg) cells. Thus, inducing a peripheral Treg response in older individuals might be a feasible strategy for increasing Treg cell levels until we have more plausible options for thymic rejuvenation. FOXP3 transcription factor (TF) is the most important regulator of Tregs and age-associated immunosenescence. FOXP3 TF expression is regulated by chemical modification by sirtuin (sirt) and histone deacetylases, in particular Sirt1 and HDAC9 [110, 111]. Interestingly, NAD is essential for sirtuin action. Therefore, it is plausible that NAD and NAD-related compounds such as NR and NMN, which are under investigation as therapeutic interventions that increase serum and cellular NAD levels, also act via Sirt1 along the FOXP3 and Treg axis, and play a role in immunosenescence and “inflammaging”.

A brief summary of the conventional and geroprotective and senoremediative strategies for patients 60 or older is provided in Table 1.

**Table 1:** The benefits and risks of conventional and geroprotective strategies for patients 60 or older.

**Intervention**	**Mechanism**	**Validation**	**Expected Benefit**	**Side Effects and Risks**
**Conventional Antiviral Strategies**
Vaccines	Contains antigenic material that mounts an immune response which is then augmented on exposure to the virus and offers protection against the disease.	Major global antiviral strategy to prevent new infective cases and fight epidemics. Viral illnesses eradicated/controlled due to previous successful vaccination programs.	Vaccinated people do not develop an infection or develop a milder infection. Reduces the number of new cases and the progression of the epidemic. Lower effectiveness in the elderly.	COVID-19 is a gerolavic infection. Efficacy of the vaccine will likely be significantly reduced due to immunosenescence and multimorbidity. Possible immunogenicity and mild viral prodrome symptoms as a result of vaccination.
Targeted Antibodies for COVID-19	Antibodies of serum of recovered individuals. Antibodies targeting specific SARS-CoV-2 proteins.	Successfully trialed in other viral diseases including Ebola.	Reduction in disease severity and lethality in exposed individuals.	Risk of systemic immune reactions and certain blood borne infections.
Targeted Small Molecule Drugs for SARS-CoV-2	Selective small molecule inhibitors targeting SARS-CoV-2 proteins such as 3C-like protease.	Multiple examples from Influenza. Neuraminidase and endonuclease inhibitors.	Reduction in disease duration, severity, and lethality in exposed individuals.	Mild side effects such as nausea, vomiting, diarrhea, etc.
Symptomatic Treatments	Non-steroidal anti-inflammatory drugs (NSAIDs), antibacterials, pain management.	Multiple clinical trials, common use.	Reduction in severity of disease.	Mild side effects such as nausea, vomiting, diarrhea, etc.
**Geroprotective and Senoremediation Strategies**
Rapalogs	mTOR inhibition.	Mouse, monkey, and human phase 2a studies in aging, over 95 phase 3 and phase 4 studies in humans for multiple diseases.	Infectivity: reduced; significant reduction when administered with vaccines. Severity: reduced. Lethality: reduced. Other benefits: Improved immune function that might increase the probability of increased longevity.	Risk of pancytopenia and potentially fatal infection risk. Risk of nephrotoxicity although milder side effect profile reported in preliminary studies.
Metformin	mTOR inhibition. Mitochondrial complex 1 inhibition. Reduction of hepatic gluconeogenesis.	Long-term use in humans. 1^st^ line treatment for type 2 diabetes, leading to weight loss and improved glucose metabolism. Improved fertility in polycystic ovarian syndrome.	Reduction in cardiovascular events, cancer, dementia, and mortality.	Diarrhea in 20% of patients. Contraindicated in patients with eGFR <15 and reduced dose required if eGFR <30 due to risk of lactic acidosis.
NAD Boosters	Sirtuin Activation. PARP efficacy augmentation. Improvement in cellular metabolism and repair mechanisms.	Reduction in blood pressure. Improvement in exercise capacity.	Reduction in immunosenescence, thus reduced infection risk and improved vaccine response. Improved endothelial function and reduced cardiovascular disease risk, potential increased lifespan.	No increase in cellular NAD levels with supplementation and no improvement in immune function. Side effects of nicotinic acid include headache, skin flushing and dizziness. Uncertain effects on circulating inflammatory cytokines.
Senolytics	Reduction in anti-apoptotic senescent inactive cells. Multiple drug classes mentioned with varying disease mechanisms.	Reduced adipose tissue senescent cell burden in a short human trial. Improvement in pulmonary fibrosis treatment.	Reduction in senescent cell burden and thus chronic sterile inflammation; reduction of chronic disease burden.	Removal of quiescent stem cells and depletion of stem cell reserve. Drug-specific side effects. Possible elimination of pre-senescent cells leading to cardiovascular, kidney, liver, and CNS damage.
Caloric Restriction and Intermittent Fasting	Promotion of autophagy, metabolic flexibility. Increase in beta hydroxybutyrate.	Human studies showing improvement in weight, glucose levels, fasting insulin and plasma lipids that go beyond weight loss.	Activated self-defenses, reduction in body weight, improvement of insulin resistance, thus reduced cardiovascular risks and certain cancer type risks.	Sarcopenia, osteoporosis. Risk of nutritional deficiencies and worsening of eating-disorders.
Growth Hormone (GH)	Action via GH receptor and IGF-1 axis.	Used in humans with GH deficiency. Improvement in anabolic function and increase in muscle mass.	Prevention of thymus degeneration and increase in immune function. Improved mood and body composition.	Increased cancer risk. Type 2 diabetes. Connective tissue proliferation.
FOXP3+ activation	One of the key mediators of immune regulation, Tregs express high levels of FOXP3. FOXP3 expression is essential for Treg development and function.	Tregs were unable to develop in a mouse receiving FOXP3-deficient progenitor cells from another animal and retroviral expression of FOXP3 in human T-cells enabled the conversion of non-regulatory naïve T-cells into a Treg-like phenotype.	FOXP3 is a Treg -specific transcription factor essential for Treg functions. Rapamycin facilitates the expansion of functional CD4+CD25+FOXP3+ Treg cells.	Treg cells are a heterogeneous population and their stability and plasticity under inflammatory conditions may pose serious problems for their clinical usage. No therapy yet available to modulate this cell subgroup safely in humans.

### On the timing of geroprotective interventions

**Figure 4:**
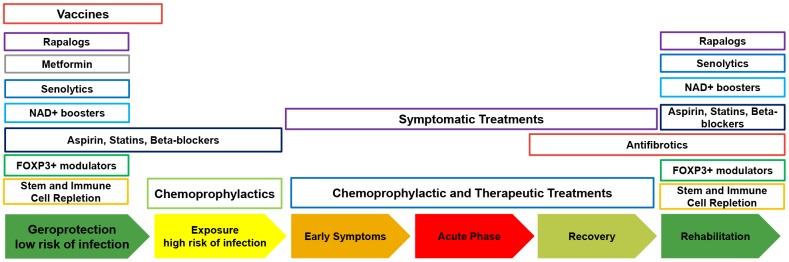
**The timing of the administration of geroprotectors for prevention, treatment and rehabilitation of gerolavic respiratory diseases.**

While there are decades of clinical evidence supporting the use of rapalogs, such as sirolimus, everolimus, and metformin, substantial meta-analysis and additional clinical trials must be conducted to understand the population-level and individual effects of these drugs taken as single agents and in combination in the context of gerolavic diseases. In this paper I propose conducting clinical trials on these known geroprotectors as a preventative measure before patients are exposed to disease (Figure 4). In the case of COVID-19 as the number of cases worldwide increases, meta-analysis of infection rates, severity, and lethality should be performed rapidly to evaluate the effects of geroprotectors, with particular focus on rapamycin. Since COVID-19 engages the immune system to damage the lungs, it may be entirely plausible that the immunomodulatory properties of rapamycin may go beyond prevention and may provide an effective treatment option. However, this hypothesis must be validated using meta-analysis before being proposed for a clinical trial.

As COVID-19 causes substantial lung damage, antifibrotics, senolytics and other geroprotectors may be explored in clinical trials to assist in patient recovery to prevent a reduction in respiratory function.

### Using biological aging clocks as markers of immunosenescence

Since senescence varies among individuals, a person’s chronological age is not as important as their biological age. For several years, scientists have sought accurate aging biomarkers that may predict an individuals’ biological age and, independently of immunosenescence, their risk of morbidity and mortality. These biomarkers, or “clocks”, could then be used to test for the effectiveness of proposed geroprotective treatments and as surrogate markers in anti-aging clinical trials. While there are no reliable aging clocks to evaluate immunosenescence and inflamaging [112], these biomarkers may be rapidly developed using historical data. At present, age clocks trained on clinical blood tests [113], transcriptomic [114] and proteomic data [115], methylation clocks [46, 116], microbiomic clocks [117] and other clocks have been described. Recent advances in artificial intelligence have enabled the development of multi-modal multi-omics age-predictors, able to learn complex non-linear patterns and extract the most important features [113, 118]. None of these currently have robust clinical validation and cannot yet serve as companion biomarkers for geroprotective and anti-aging interventions intended to ameliorate the population-level effects of infectious diseases during flu seasons and pandemics. We call for rigorous clinical validation and further development of biological aging clocks that could, in the future, allow us to measure the effectiveness of the numerous speculative geroprotective and senoremediative interventions described herein.

## DISCUSSION

SARS-CoV-2 is a betacoronavirus that causes respiratory illness, and is genetically most similar to SARS-CoV, the betacoronavirus that caused the SARS epidemic of 2003. The Middle East respiratory syndrome (MERS) epidemic was also caused by a betacoronavirus that induces severe respiratory illness. Both SARS and MERS were contained before they became pandemics, and much of what we predict about the trajectory of COVID-19 comes from what we learned about SARS and MERS [24]. Theoretically, treatments found to be effective against SARS and MERS are the most promising starting points for treatments likely to be effective against SARS-CoV-2.

The SARS outbreak of 2002 was rapidly contained, and no new cases have been reported since 2004 [119]. Since the scale of the outbreak did not provide any commercial benefit for the pharmaceutical industry to develop effective drugs for SARS, much of the discovery efforts stopped after the epidemic. When the news of SARS-CoV-2/COVID-19 emerged in early January 2020, it was difficult to justify a business case for small biotechnology companies to allocate resources to the effort. By January 28^th^, however, Insilico Medicine allocated resources to generate and test small molecules against the SARS-CoV-2 3C-like protease [120, 121]. As the scale of the current COVID-19 pandemic remains uncertain, it is still difficult to justify allocating scarce company resources to full-scale drug discovery and drug development programs, which may cost tens or even hundreds of millions of dollars [122]. Multiple biotechnology companies are in the same situation and will not be able to proceed without substantial backing from government agencies, non-profit organizations, or bigger pharmaceutical companies. However, given the gerophilic and gerolavic nature of COVID-19, strategies targeting age-associated pathologies and immunosenescence, which could decrease the comorbidity, infection rates, severity, and lethality of the disease, will remain commercially-viable even when the pandemic subsides. In addition, respiratory infections are now the third leading cause of death in the world, following cardiac disease and stroke [123], further justifying the need for these interventions.

Considering the gerolavic nature of COVID-19, where the majority of the seriously affected population is older than 60, classical prevention and treatment strategies may not be effective. Given the severity and lethality of the pandemic, even healthcare systems in developed countries will find it challenging to cope with the increased disease burden and hospital needs. Conventional approaches to prevention such as vaccines are much needed, but even these do not offer complete protection in the elderly due to multi-morbidity and age-related immune declines. Therefore, interventions that enable immunocompromised elderly to mount an immune response to newly developed vaccines are necessary to help eradicate the disease and reduce the associated mortality.

To avoid substantial loss of life and quality of life, primarily among the elderly and vulnerable populations, governments and healthcare systems should investigate preventative and intervention strategies stemming from recent advances in aging research. As discussed in this paper, small clinical studies have shown that several geroprotective and senoremediative interventions, such as treatment with sirolimus and rapalogs, can induce immunopotentiation, increase resistance to infection, and reduce disease severity in the elderly, without severe side effects. Serendipitously, during the revision of this article, another group utilizing computational approaches proposed using melatonin and sirolimus (rapamycin) in combination to treat the COVID-19 infection outside the context of geroprotection [124].

Many of these predicted geroprotectors are available as supplements; however, no meta-analysis or metaclinical trials have been performed at scale to evaluate their effectiveness. The COVID-19 pandemic highlights the paucity of clinical trials on the effects of dietary supplements and drugs on aging and immunosenescence. The existence of pseudoscience and anecdotal promotion in the supplement industry does not mean that protective compounds do not exist. Dietary supplement vendors and pharmaceutical companies need to actively engage in preclinical and clinical research to evaluate the effectiveness of the currently available products on immunosenescence and aging.

## CONCLUSIONS

This paper is not intended to encourage the use of rapalogs or other potential geroprotectors during the COVID-19 pandemic. It may be possible that some of the potential geroprotectors described in this paper are harmful to the elderly after infection, and may actually increase disease severity and lethality. However, it may be possible to conduct clinical trials on the efficacy of geroprotectors previously tested in human clinical trials in treating COVID-19 and other gerophilic and gerolavic infections.

To combat the growing COVID-19 pandemic, researchers have united globally to tackle a disease that is impacting lives and healthcare systems around the world. After carefully analysing preliminary data, we suggest that COVID-19 has a gerophilic and gerolavic profile, being more infectious and more severe in the elderly. In this paper, we review the current literature on speculative aging reversal treatments, such as experimental geroprotective strategies using everolimus (RAD001) and sirolimus (rapamycin). We summarize the current possible interventions and identify the lack of clinical evidence to support their immediate use with the aim of encouraging further, more rigorous reviews of geroprotective compounds such as rapalogs, metformin, senolytics, and conventional and investigational NAD+ boosters. We also suggest that further clinical studies should be carefully designed and adequately powered to determine if these interventions might provide clinical benefit as adjuncts to vaccines and antiviral treatments by acting as immune response potentiators. Lastly, as with many other diseases, COVID-19 is more common and severe in elderly populations, and we thus invite further research and clinical validation in the field of biological aging clocks. These markers could potentially be used in the future to measure and analyze immunosenescence and the efficacy of interventions claimed to slow down or reverse age-related immune decline.

### Disclaimer and limitations

This perspective is of a highly speculative nature presented during the time of a global COVID-19 pandemic. It is intended for a professional audience to stimulate ideas and aid the global efforts of the scientific community to develop effective new treatments for this disease. This article does not represent medical advice or recommendations to patients. There is no clinical evidence to support the use of the treatments described in this article for this indication and the authors do not advise anyone to self-administer these drugs as COVID-19 prevention or treatment. Furthermore, this perspective is based on the limited data from the first weeks of the COVID-19 outbreak. The demographic distribution of the infected and diseased may change and differ in different countries with different social customs and different ethnicities. The media should exercise caution and seek expert medical advice for interpretation when referring to this article to avoid misinterpretation or unsafe messages being delivered to the community amidst exceptional coverage of this disease in the media at present.
